# (1→3)-β-D-glucan in patients with pulmonary aspergilloma

**DOI:** 10.1080/09629359791631

**Published:** 1997-11

**Authors:** Kazumi Yuasa, Hajime Goto

**Affiliations:** 1 Department of Pulmonary Disease Tokyo Metropolitan Komagome General Hospital 3-18-22 Honkomagome Bunkyou-ku Tokyo 113 Japan

## Abstract

To elucidate the role of (1→3)-β-D-glucan in pulmonary aspergilloma, serum concentrations of (1→3)-β-D-glucan were measured repeatedly for as long as 10 months in eight patients. In four patients with inactive disease, concentrations of (1→3)-β-D-glucan were in the normal range.The concentrations of (1→3)-β-D-glucan increased in two patients, although the disease was inactive. This increase might show the earliest stage of the invasive process of the disease. In two other patients with active disease, (1→3)-β-D-glucan increased. Other parameters, such as galactomannan, immunodiffusion and a radio-allergosorbent test, as well as inflammatory m arkers such as C-reactive protein and the leukocyte count, did not show any consistent tendency in regard to the activity of the disease. Thus, a (1→3)-β-D-glucan assay may add valuable data for evaluating the disease activity and understanding the disease process of pulmonary aspergilloma.


Mediators of Inflammation, **6**, 285–287 (1997)

### (1→3)-β-D-glucan in patients with pulmonary aspergilloma

**Kazumi Yuasa**[CA] **and Hajime Goto**

Department of Pulmonary Disease, Tokyo Metropolitan Komagome General Hospital, 3-18-22 Honkomagome, Bunkyou-ku, Tokyo 113, Japan

[CA]Corresponding author
Tel: +81 3 3823 2101
Fax: +81 3 3824 1552

**To elucidate the role of (1→3)-β-D-glucan in pulmonary aspergilloma, serum concentrations of (1→3)-β-D-glucan were measured repeatedly for as long as 10 months in eight patients. In four patients with inactive disease, concentrations of (1→3)-β-D-glucan were in the normal range. The concentrations of (1→3)-β-D-glucan increased in two patients, although the disease was inactive. This increase might show the earliest stage of the invasive process of the disease. In two other patients with active disease, (1→3)-β-D-glucan increased. Other parameters, such as galactomannan, immunodiffusion and a radio-allergosorbent test, as well as inflammatory markers such as C-reactive protein and the leukocyte count, did not show any consistent tendency in regard to the activity of the disease. Thus, a (1→3)-β-D-glucan assay may add valuable data for evaluating the disease activity and understanding the disease process of pulmonary aspergilloma.**

**Keywords:** Inflammatory markers

### Introduction

In a previous study, we measured serum (1→3)-β-D-glucan in patients with pulmonary aspergillosis and found that (1→3)-β-D-glucan increased in some cases of pulmonary aspergilloma, in which the fungus was considered not to have invaded the surrounding tissue or blood stream [1].

In the present study, to elucidate the role of (1→3)-β-D-glucan in pulmonary aspergilloma, serum concentrations of (1→3)-β-D-glucan were measured repeatedly for as long as 10 months in eight patients.

### Materials and methods

### Subjects

Eight patients with pulmonary aspergilloma were enrolled in the study (Table 1). Of the eight patients, the disease was inactive in six. In patient 7, hemosputum was frequently observed throughout the follow-up period, and in patient 8, the fungus ball steadily increased in size. The disease was judged to be active in these two patients. Itraconazole (ITCZ) at 100–200 mg/day, was administered throughout the follow-up period to all but patient 7.

### Clinical evaluation

The patient's physical condition, a chest X-ray and blood tests (leukocyte count, C-reactive protein) were examined once a month in each patient to evaluate the activity of the disease.

### Measurement of (1→3)-β-D-glucan

The concentrations of (1→3)-β-D-glucan were measured following the method described by Obayashi *et al.* [2,3] and Morita *et al.* [4]. With this method, the cut-off value for (1→3)-β-D-glucan was set at 20 pg/ml [5].

### Measurement of other *Aspergillus*-related substances

Anti-*Aspergillus* immunoglobulin (Ig)E antibody was measured by a radio-allergosorbent test (>0.34 U/ml was positive). The presence of *Aspergillus*-specific antibodies in sera was examined by immunodiffusion. The *Aspergillus*-specific cell-surface component galactomannan was assayed with Pastorex Aspergillus (Sanofi Diagnostics Pasteur, Genk, Belgium). The sensitivity of the test was 15 ng/ml of galactomannan [6].

### Results and discussion

On the basis of the concentration of (1→3)-β-D-glucan and the clinical activity of pulmonary aspergilloma, the patients were classified into three groups: group 1, normal (1→3)-β-D-glucan with inactive disease; group 2, increased (1→3)-β-D-glucan with inactive disease; and group 3, increased (1→3)-β-D-glucan with active disease.

Group 1 consisted of patients 1–4 (Table 2). In this group, the values of galactomannan, immunodiffusion, radio-allergosorbent test and C-reactive protein were variable in each patient, and there were no significant correlations among these values.

Patients 5 and 6 were classified in group 2 (Table 3). In this group, (1→3)-β-D-glucan exceeded 20 pg/ml. However, there were no obvious signs of active disease. Other parameters, such as galactomannan, immunodiffusion, the radio-allergosorbent test and

© 1997 Rapid Science Publishers

**Table 1.** Details of all study patients

| Patient | Age (years) | Sex | Diagnosis | Treatment | Initiation of treatment |
|---|---|---|---|---|---|
| 1 | 60 | Male | Aspergilloma | ITCZ at 150 mg/day | 21 Nov 1995 |
| 2 | 78 | Male | Aspergilloma | ITCZ at 200 mg/day | 14 Apr 1995 |
| 3 | 78 | Male | Aspergilloma | ITCZ at 100 mg/day | 11 Jan 1994 |
| 4 | 67 | Female | Aspergilloma | ITCZ at 150 mg/day | 05 Dec 1994 |
| 5 | 67 | Male | Aspergilloma | ITCZ at 200 mg/day | 28 Feb 1995 |
| 6 | 67 | Male | Aspergilloma | ITCZ at 100 mg/day | 28 Sep 1994 |
| 7 | 79 | Male | Aspergilloma | | |
| 8 | 83 | Male | Aspergilloma | ITCZ at 150 mg/day | 23 Aug 1995 |

ITCZ, itraconazole.

**Table 2.** Follow-up study of group 1

| | Leukocytes (count/μl) | CRP (mg/dl) | RAST (U/ml) | Immuno-diffusion | Galacto-mannan | Glucan (pg/ml) |
|---|---|---|---|---|---|---|
| **Patient 1** | | | | | | |
| 08 Nov 1995 | 7400 | 8.7 | 4.2 | + | – | 13.4 |
| 11 Nov 1995 | 6900 | 4.2 | ND | ND | ND | 4.9 |
| 04 Dec 1995 | 6600 | 5.0 | 4.12 | + | – | 6.3 |
| 22 Jan 1996 | 8300 | 4.9 | 3.42 | + | – | 11.7 |
| 19 Feb 1996 | 6400 | 5.1 | 3.99 | + | – | 13.6 |
| 18 Mar 1996 | 7500 | 5.0 | 2.96 | + | – | 13.6 |
| **Patient 2** | | | | | | |
| 13 Jun 1995 | 4700 | 0.8 | 0.37 | + | – | 9.0 |
| 07 Aug 1995 | 4900 | 0.9 | <0.34 | + | – | 19.3 |
| 04 Sep 1995 | 4500 | 1.1 | <0.34 | + | – | 15.3 |
| 03 Oct 1995 | 5200 | 1.1 | 0.37 | + | – | 5.2 |
| 30 Oct 1995 | 4800 | 0.6 | 0.46 | + | – | 14.3 |
| 27 Nov 1995 | 5400 | 0.4 | 0.69 | + | – | 4.5 |
| 26 Dec 1995 | 7700 | 0.4 | 0.50 | + | – | 11.2 |
| 22 Jan 1996 | 5500 | 0.9 | 0.49 | + | – | 12.0 |
| 19 Feb 1996 | 6800 | 0.6 | 0.53 | + | – | 14.7 |
| 18 Mar 1996 | 5800 | 1.4 | 0.56 | + | – | 3.0 |
| **Patient 3** | | | | | | |
| 16 May 1995 | 7000 | 0.4 | 1.46 | + | ND | 6.8 |
| 20 Jun 1995 | 5300 | 0.6 | 1.54 | ND | – | 5.6 |
| 11 Jul 1995 | 6500 | 1.0 | 1.21 | ND | – | 0.0 |
| 08 Aug 1995 | 5600 | 0.7 | 1.28 | + | + | 1.3 |
| 12 Sep 1995 | 6600 | 0.5 | 1.37 | ND | – | 0.8 |
| 20 Oct 1995 | 5900 | 0.4 | 1.58 | ND | – | 1.6 |
| 14 Nov 1995 | 7100 | 0.6 | 2.16 | + | – | 4.2 |
| 12 Dec 1995 | 5500 | 1.2 | 2.22 | ND | – | 6.0 |
| 16 Jan 1996 | 5300 | 0.4 | 1.85 | ND | – | 19.2 |
| 13 Feb 1996 | 5400 | 0.9 | 2.06 | + | + | ND |
| 12 Mar 1996 | 5300 | 0.7 | 1.69 | ND | – | 15.7 |
| **Patient 4** | | | | | | |
| 22 May 1995 | 6300 | 0.0 | 0.50 | + | + | 12.2 |
| 19 Jun 1995 | 7100 | 0.1 | 0.48 | + | + | 6.1 |
| 17 Jul 1995 | 7200 | 0.0 | 0.54 | + | + | 12.0 |
| 14 Aug 1995 | 7100 | 0.0 | ND | ND | ND | 8.8 |
| 25 Sep 1995 | 7100 | 0.0 | 0.43 | + | + | 4.6 |
| 23 Oct 1995 | 6900 | 0.1 | 0.35 | + | +/– | 7.4 |
| 20 Nov 1995 | 8700 | 1.2 | 0.82 | + | + | 5.8 |
| 18 Dec 1995 | 6400 | 0.2 | 0.77 | + | + | 14.9 |
| 08 Jan 1996 | 5700 | 0.1 | 0.79 | + | + | 6.5 |
| 11 Mar 1996 | 6100 | 0.1 | 0.69 | + | + | 6.7 |

CRP, C-reactive protein; RAST, radio-allergosorbent test; ND, not determined.

C-reactive protein, were dissociated from each other. Thus the reason why there appeared to be no obvious signs of disease may be that *Aspergillus* had just started to invade the surrounding tissue and had not progressed enough to be detectable by conventional methods. However, further follow-up studies are required to verify this hypothesis.

Group 3 comprised patients 7 and 8 (Table 4). Patient 7 had complained of frequent hemosputum since 1979, when he was diagnosed as suffering from pulmonary aspergilloma. In this case, values of (1→3)-β-D-glucan and radio-allergosorbent test were elevated. Galactomannan was seldom detected, however. In patient 8, the fungus ball showed steady growth until 1995, when the patient was enrolled in the study. In this case, to elucidate the day-to-day variation, the concentrations of (1→3)-β-D-glucan were measured daily for 2 weeks. The size of the fungus ball remained

**Table 3.** Follow-up study of Group 2

| | Leukocytes (count/µl) | CRP (mg/dl) | RAST (U/ml) | Immuno-diffusion | Galacto-mannan | Glucan (pg/ml) |
|---|---|---|---|---|---|---|
| **Patient 5** | | | | | | |
| 22 Jun 1995 | 5800 | 1.6 | <0.34 | + | – | 7.2 |
| 17 Aug 1995 | 4900 | 3.1 | <0.34 | + | – | 18.8 |
| 14 Sep 1995 | ND | ND | <0.34 | + | – | 22.5 |
| 09 Nov 1995 | 7300 | 2.6 | ND | ND | ND | 22.4 |
| 07 Dec 1995 | 6000 | 2.9 | ND | + | – | 17.1 |
| 08 Feb 1996 | 6700 | 2.7 | ND | + | – | 21.3 |
| 27 Feb 1996 | 7400 | 3.0 | ND | ND | ND | ND |
| 14 Mar 1996 | ND | ND | ND | ND | ND | 21.6 |
| **Patient 6** | | | | | | |
| 18 May 1995 | 9500 | 12.7 | 1.77 | – | – | 21.0 |
| 15 Jun 1995 | 8100 | 3.7 | 1.44 | ND | – | 13.8 |
| 14 Jul 1995 | 9300 | 3.6 | 1.45 | – | – | 10.8 |
| 25 Aug 1995 | 7700 | 5.5 | 0.89 | ND | – | 26.0 |
| 22 Sep 1995 | 6500 | 3.8 | 1.16 | ND | – | 27.4 |
| 20 Oct 1995 | 9100 | 3.0 | 1.60 | – | – | 28.7 |
| 24 Nov 1995 | 7800 | 2.7 | 1.70 | ND | – | 37.3 |
| 22 Dec 1995 | 8200 | 5.0 | 2.10 | ND | – | 26.8 |
| 19 Jan 1996 | 5000 | 2.5 | 1.91 | – | – | 22.5 |
| 16 Feb 1996 | 4600 | 6.4 | 2.13 | ND | – | 14.2 |
| 15 Mar 1996 | 7600 | 4.2 | 1.85 | ND | – | 24.6 |

CRP, C-reactive protein; RAST, radio-allergosorbent test; ND, not determined.

**Table 4.** Follow-up study of Group 3

| | Leukocytes (count/µl) | CRP (mg/dl) | RAST (U/ml) | Immuno-diffusion | Galacto-mannan | Glucan (pg/ml) |
|---|---|---|---|---|---|---|
| **Patient 7** | | | | | | |
| 26 May 1995 | 5500 | 2.6 | 10.12 | ND | – | 60.1 |
| 30 Jun 1995 | 6200 | 2.4 | 9.38 | ND | – | 59.3 |
| 28 Jun 1995 | 6500 | 3.0 | 11.20 | + | + | 55.0 |
| 25 Aug 1995 | 6900 | 3.1 | 12.95 | ND | + | 52.1 |
| 22 Sep 1995 | 5500 | 2.4 | 12.37 | ND | – | 54.1 |
| 20 Oct 1995 | 6200 | 4.3 | 12.71 | + | – | 56.9 |
| 14 Nov 1995 | 5500 | 3.5 | 20.92 | ND | – | 41.8 |
| 19 Dec 1995 | 6700 | 3.3 | 18.45 | ND | – | 33.6 |
| 16 Jan 1996 | 6500 | 3.1 | 15.81 | ND | – | 24.6 |
| 20 Feb 1996 | 10 900 | 2.4 | 21.60 | ND | – | 47.6 |
| **Patient 8** | | | | | | |
| 31 Jul 1995 | 5800 | 0.2 | <0.34 | – | – | ND |
| 23 Aug 1995 | ND | ND | ND | ND | ND | 42.0 |
| 24 Aug 1995 | ND | ND | ND | ND | ND | 28.2 |
| 25 Aug 1995 | ND | ND | ND | ND | ND | 32.4 |
| 28 Aug 1995 | ND | ND | ND | ND | ND | 32.4 |
| 29 Aug 1995 | ND | ND | ND | ND | ND | 35.1 |
| 30 Aug 1995 | 5200 | 0.1 | ND | ND | ND | 36.0 |
| 31 Aug 1995 | ND | ND | ND | ND | ND | 34.5 |
| 04 Sep 1995 | ND | ND | ND | ND | ND | 16.1 |
| 05 Sep 1995 | ND | ND | ND | ND | ND | 11.1 |
| 07 Sep 1995 | 5100 | 0.2 | ND | ND | ND | 11.0 |

CRP, C-reactive protein; RAST, radio-allergosorbent test; ND, not determined.

unchanged and (1→3)-β-D-glucan values were normalized during the period.

In contrast to the prevalent notion, increased (1→3)-β-D-glucan values in this group might imply that *Aspergillus* in the lesion of aspergilloma can invade surrounding tissue and/or the bloodstream, though the invasion is radiologically undetectable. The prompt decline in serum (1→3)-β-D-glucan in patient 8 may support this concept.

In summary, the data presented here suggest that the (1→3)-β-D-glucan assay may add valuable data for evaluating the disease activity as well as for understanding the disease process of pulmonary aspergilloma.

### References

1. Yuasa K, Goto H, Iguchi M, Okamura T, Ieki R: Evaluation of the diagnostic value of the measurement of (1→3)-β-D-glucan in patients with pulmonary aspergillosis. *Respiration* 1996, **63**:78–83.
2. Obayashi T, Tamura H, Tanaka S, *et al.*: A new chromogenic endotoxin-specific assay using recombined limulus coagulation enzymes and its clinical applications. *Clin Chim Acta* 1985, **149**:55–65.
3. Obayashi T, Yoshida M, Tamura H, Aketagawa J, Tanaka S, Kawai T: Determination of plasma (1→3)-β-D-glucan: a new diagnostic aid to deep mycosis. *J Med Vet Mycol* 1992, **30**:275–280.
4. Morita H, Urashima Y, Tanaka S, Aketagawa J, Tamura H, Kumazawa Y: Simultaneous estimation of endotoxin and (1→3)-β-D-glucan with an automated kinetic chromogenic assay using Wellreader. *J Antibact Antifung Agents* 1996, **24**:467–475.
5. Obayashi T, Yoshida M, Mori T, *et al.*: Plasma (1→3)-β-D-glucan determination in the diagnosis of invasive deep mycosis and fungal febrile episodes. *Lancet* 1995, **345**:17–20.
6. Stynen D, Sarfati J, Goris A, *et al.*: Rat monoclonal antibodies against *Aspergillus* galactomannan. *Infect Immun* 1992, **60**:2237–2245.
